# Collectanea Medica: Consisting of Anecdotes, Facts, Extracts, Illustrations, &c.

**Published:** 1820-11

**Authors:** 


### COLLECTANEA MEDICA:

CONSISTING OF

ANECDOTES, FACTS, EXTRACTS, ILLUSTRATIONS, &c.

*Relating to the History or the Art of Medicine, and the Auxiliary Sciences.*

---

Floriferis ut apes in saltibus omnia libant,

Omnia nos itidem depascimur aurea dicta.

---

### *Extracts of the Report from the Select Committee of the House of Commons on the Doctrine of the Contagion of the Plague, in* 1817.

*Martiis, 29° die Aprilis,* 1819.

SIR JOHN JACKSON, Baronet, in the Chair.

Mr. EDWARD HAYES, called in; and Examined.

HAVE you resided long in a plague-country?—Yes; I was born at Smyrna, and resided there nearly all my life. I have resided there nearly forty-four years, from the time I was born.

During that period have you seen frequent occurrences of the plague?—Very frequent occurrences of it.

Did you avoid it yourself?—We always do.

By what means?—We shut ourselves up from communication with the town, and only communicate through purveyors.

Do those purveyors have communication with persons who are infected?—Yes; but they are generally people who have had the plague themselves.

Had you the plague?—No. It was once in our family, and I thought I had symptoms of it, but it was not the plague. It had got into our house, and several individuals died of it, and among others a relation.

Notwithstanding all was shut up in your house?—Yes: that sometimes happens; but we can almost always trace it to some imprudence of our domestics.

Will you explain what imprudence?—The receiving any articles without putting them through a purification of air, water, or a fumigation.

Have you known instances of persons having communication with persons who were afflicted with the plague, without catching the infection themselves?—Very often.

As often as on the contrary?—No, not by any means.

Do you consider the plague to be absolutely contagious?—Certainly: that is to say, the atmosphere must be in a state when it will spread a great deal more than at other times.

What become of the clothes of a person who dies of the plague?—When an European dies of the plague, we generally destroy the clothes: I mean such as we cannot wash. But, when the natives die of the plague, the clothes are sold again; that is, *until lately*. They now discover their folly, and begin to take the same precautions that they do with respect to European's clothes: at least with regard to the higher order of Turks; but it is by no means a general custom.

Then do the Turks make no hesitation in putting on the garments of a person who has died of the plague?—The greatest part of them do not.

Nor in sleeping in the same places?—No; but that arises, in a great degree, from their being predestinarians.

When does the plague generally subside in your country?—When the great cold or the great heat destroys it. It is erroneously supposed that it must cease about the 24th of June, because the 24th of June is St. John's day; but it is quite absurd to suppose that, because I have seen it cease sometimes before; and I have seen it continue sometimes as late as July or August, when the great heats destroy it.

And when do the great heats usually destroy it?—The virulence of the disorder is almost invariably over by August.

When does it begin?—In March or April.

Do you ship goods promiscuously, whether the plague rages or not?—Yes.

To all parts of Europe?—Yes.

Do you imagine that goods shipped during the time the plague is raging, convey the contagion?—Certainly; and that every country that receives those goods receives in a degree the contagion of the plague.

To what do you suppose it may be attributed, that England has not the plague from that circumstance?—To the climate, to the length of the voyage, and the exposure of the goods to the air.

If your goods are imported into this country, how comes it that the expurgators of those goods do not catch the plague?—From the same circumstances, perhaps, as that I have seen persons not catch the plague, though attending upon plague people. It may arise from the state of the body: there has been plague in the lazarettos in other countries.

Do you consider the lazarettos of any use in this country?—They are very imperfect; and are by no means adequate to keeping the plague out of the country.

Do you think they are of some use?—Certainly they are of some use: the length of time that persons are obliged to stay there must be of use.

Are there any quarantine-establishments in Holland?—Yes; and vessels that have quitted the Levant at the time the plague prevails will not be permitted to enter their ports.

You have stated, that the purveyors who carry on the communication between the natives and Europeans during the existence of plague are generally persons who have had the plague: are individuals who have had the plague once less likely to have it again?—If they have had the plague in the severe stage once, and, during a period of plague afterwards, if they take every precaution, and keep a proper regimen, they are much less liable to have it, though they are by no means certain of being exempted, because I have known people to have it ten times.

You have stated, that the plague frequently makes its way into European families, from the imprudence of domestics: do you speak from your own knowledge of any case in which the plague has been introduced, by the introduction of goods of any description into an European family?—Certainly; repeatedly.

From your own knowledge?—Yes, dear-bought knowledge; for it was brought into our family in that way.

In what way?—By servants bringing stockings, or any thing of that kind, into the house, without their having undergone a proper purification.

Are you, however, satisfied that it was introduced by those goods alone, or might it not have been by contact with persons affected by plague?—I have known cases where it has been introduced by goods, and also by walking out, and having communication with individuals who had the plague without our knowing it.

But I wish to put it to you, whether, within your own knowledge, any case has occurred from which you are satisfied in your own mind that it was introduced solely by goods, without contact?—Repeatedly; I have known many obvious cases of that sort.

Is it your opinion that goods that have been touched by infected persons will retain that infection for any length of time?—If those goods were closely packed up, they would retain it for a long time; but, if the air comes to them, it destroys it. I am however persuaded, that plague might be kept in goods or boxes closely shut up for many years.

What species of goods are you of opinion are most liable to retain and convey the infection?—All wools, and any linen; any cotton goods. Goats-wool is particularly adapted to retain the infection, on account of the way in which it is prepared.

Are goats themselves subject to the plague?—No.

Are animal substances more likely to retain and convey the infection than vegetable substances?—Certainly.

But cotton goods are liable to carry the infection?—Yes.

Are there any substances which are not liable to retain the infection?—Yes, some substances will not retain it; a stone, for instance, does not: but I should be very sorry to put my hand upon a stone immediately after it had been touched by a person afflicted with the plague.

You say that the inhabitants of Smyrna are in the habit of using many precautions against the plague: have you ever heard of inoculation for the plague?—I have known one or two instances; but, as the patients are liable to take it again, it is of very little use.

Have you ever known the plague communicated by inoculation?—In one or two instances.

Has that inoculation caused the plague?—Yes.

Is the cow-pock known at Smyrna?—Yes.

Has it ever been tried as a preventive against the plague?—Not that I know of. It is unfortunate that the consequences of the disease are so rapidly destructive, that few medical men ever made it a point to study the nature of the disease: there have been several doctors attempted it, but all of them have fallen victims. One doctor, whose name I forget, was sent to Smyrna by Catherine of Russia, and he waited with great patience for a considerable time while no plague was there: he waited until a case of plague occurred; he went into the hospital, and he died in three days afterwards. His mode of attempting to cure the plague was by frictions of snow; but I have good reason to believe that friction of oil is a very good application.

Have you ever known mercury used?—It has never been used that I know of.

Did you ever know of a person going into a plague-hospital, and coming out cured?—Yes, a great many.

What sort of hospitals are they?—There are Roman Catholic hospitals, Armenian hospitals, and hospitals for Jews; and, what is a most extraordinary fact, that, even filthy as they are, they recover; but I believe that is in a great degree owing to the care that is taken of them; for there is very little care taken in the Greek hospitals.

